# Improving Quality and Appropriateness of Referrals to a Drug and Alcohol Outreach Service in a General Psychiatric Hospital

**DOI:** 10.1192/bjo.2025.10357

**Published:** 2025-06-20

**Authors:** Eileen Dempsey, Rebecca Lawrence

**Affiliations:** NHS Lothian, Edinburgh, United Kingdom

## Abstract

**Aims:** By March 2025, 90% of referrals to the Ritson Outreach Service in the Royal Edinburgh Hospital will be appropriate and contain relevant details.

**Methods:** Members of the Ritson Outreach team agreed the following referral criteria for inpatients on general psychiatric wards:

Prescribing for alcohol withdrawal and relapse prevention.

Prescribing in opioid dependence.

Prescribing in benzodiazepine dependence.

Advice on linking to community services.

Standards for referral details were also agreed: ward, referrer, contact number, reason for admission, specific request, community addictions input, patient’s awareness and views on referral, drug screen results, estimated discharge date, appropriateness according to referral criteria.

A baseline audit of referrals to the Ritson Outreach inbox from 19 March–28 August 2024 was conducted. Surveys about barriers to making appropriate referrals were gathered from the ward with the highest referral rate. A referral form including criteria and prompts for relevant details was devised. This was made available via an automatic reply from the referral email address. Referrals made following implementation of the form were re-audited for a four-week period from 8 January 2025.

**Results:** 20 referrals were included in the baseline audit.Adherence to standards: Ward 100%; Referrer 100%; Contact number 55%; Reason for admission 60%; Specific request 55%; Community addictions input 30%; Patient aware of referral 20%; Patient’s views 40%; Drug screen results 5%; Estimated discharge date 5%; Appropriate 55%.

10 surveys from the ward with the highest referral rate revealed only 10% of staff felt confident about the referral criteria and relevant details to include prior to implementation of the referral form.

In the four-week period following implementation of the referral form, 5 referrals were received via the referral mailbox. Adherence to standards: Ward 100%; Referrer 100%; Contact number 100%; Reason for admission 80%; Specific request 100%; Community addictions input 80%; Patient aware of referral 60%; Patient’s views 60%; Drug screen results 20%; Estimated discharge date 40%; Appropriate 80%.

**Conclusion:** Implementation of a referral form has begun to improve quality and appropriateness of referrals to the Ritson Outreach Service, although not yet reaching the target of 90% appropriate referrals. Further data collection is ongoing, along with measures to increase staff awareness of the referral criteria and process, such as posters in handover rooms and inclusion in resident doctor inductions.

